# Essentials of Diagnosis, Arranged in the Form of Questions and Answers

**Published:** 1900-09

**Authors:** 


ESSENTIALS OF DIAGNOSIS, arranged in the form of Questions and Answers. Prepared especially for students of medicine. By SOLOMON SOLIS-COHEN, M. D., Professor of Clinical Medicine and Therapeutics in the Philadelphia Polyclinic, etc., and AUGUSTUS A. ESHNER, M. D., Professor of Clinical Medicine in the Philadelphia Polyclinic, etc. Illustrated. Second edition revised and enlarged. Philadelphia: W. B. Saunders. 1900. [Price, $1.00.

No introduction is necessary to the excellent series of medical works known from Maine to California as Saunders's Question Compends. Every student in medicine for the past ten years has in some way or other gleaned some knowledge, some fact from these handy little volumes, which say so much in such a limited space and which lasts after it has been said. The profession also looks to this series when hurriedly in search of important information and generally finds exactly what is sought after. The volume on medical diagnosis by the two distinguished authors is up to the high standard of the series and in this second edition they have been able to enlarge, rearrange and revise, and so make this volume as complete and accurate as the present state of scientific medicine will permit.

The binding, paper and print conforms with other numbers of this series. W. C. K.

